# Modeling delay to diagnosis for Amyotrophic lateral sclerosis: under reporting and incidence estimates

**DOI:** 10.1186/1471-2377-12-160

**Published:** 2012-12-23

**Authors:** Irene Rocchetti, Domenica Taruscio, Daniela Pierannunzio

**Affiliations:** 1, ISTAT, Dipartimento per i censimenti e gli archivi amministrativi e statistici, Via Tuscolana 1788, 00173 Rome, ITALY; 2 Istituto Superiore di Sanitá, Centro Nazionale Malattie Rare, Via Giano Della Bella 34, 00161 Rome, ITALY; 3 Istituto Superiore di Sanitá, Centro Nazionale Epidemiologia, Via Giano Della Bella 34, 00161 Rome, ITALY

**Keywords:** Delay to diagnosis, Horvitz-Thompson, Incidence estimate, Rare diseases, Reverse hazard

## Abstract

**Background:**

This paper provides a strategy to obtain a reliable estimate of the incidence rate for Amyotrophic lateral sclerosis based on data from the National Registry of Rare Diseases (NRRD). In fact, unobserved cases may be due to the fact that “a long time” may intercour between the suspect of having the disease (onset) and the date the disease is diagnosed. Potential factors that may influence the probability of experiencing the event (diagnosis) conditionally on the onset (suspected) are investigated. Since we are treating rare diseases, the role of social and economic factors is not that obvious; latent as well as observed factors may influence the delay to diagnosis.

**Methods:**

We use a semiparametric estimator based on the distribution of delay to diagnosis to account for potential underreporting. In particular, we propose to adopt an Horvitz-Thompson based estimator to correct the incidence figure that can be derived for the period 2007-2009 from the NRRD, Italy.

**Results:**

The incidence estimates obtained by adopting the proposed approach are about 1 case per 100000 inhabitants and despite they let recovering a good part of underreporting, they are still far from ALS incidence international ranges between 1.5 and 2.5. However, by looking only at northern Italy, the incidence estimates we can derive are coherent with those known internationally.

**Conclusions:**

These results confirm the existence of substantial differences in reporting accuracy, and point out where the system of data collection must be improved. In particular, when reliable individual characteristics will be available, they could be employed to refine the proposed estimator.

## Background

The National Registry for Rare Diseases (in the following NRRD), see http://www.iss.it/cnmr/, includes more than 90.000 (year 2009) diagnosed cases and provides information about the date of (suspected) disease onset, the date of diagnosis and other individual details such as age, educational level, professional status. The registry has been established according to article 3, decreto ministeriale 279/2001 (see [1]), to provide information about the distribution of rare diseases in the general population. A disease is considered as rare when it affects no more than 5 individuals per 10,000 people. According to the World Health Organization (WHO), the number of known rare diseases ranges from 7,000 to 8,000.

The NRRD collects epidemiological information about the number of cases for a given pathology and has been established to study the corresponding geographical distribution, to plan health services organization, to pursue better/more efficient care pathways. Given the available information, it helps estimate the time elapsed between the onset (date of first occurrence of symptoms that can be univocally linked to the disease) and the diagnosis, which is often termed delay to diagnosis. All patients reported in the registry have experienced the event of diagnosis for a rare disease; the onset date is ascertained at the time of diagnosis.

Administrative registries may fail to answer epidemiological questions; in particular, prevalence and incidence estimates can be quite difficult to be provided. This is due to the quality of such registries: they may be incomplete, they may not provide a correct registration of the analyzed event, they may not cover the whole national territory and/or the whole time window, leading to geographical and temporal coverage problems, respectively. These issues characterize the NRRD as well: for example, only 17 Regions out of 20 (corresponding to approximately 80% of the total population) did send rare disease data to the Institute of Public Health (ISS) which is responsible for data collection; not all the presidiums in these regions may be active in collecting data. To get reliable prevalence and/or incidence rate estimates, we need to develop statistical methods to handle the problem of underreporting.

The problem of underreporting can be linked to different aspects; in our case, a high geographical variability in registration coexists with a high variability in the observed distribution of delay to diagnosis. The reasons are different: for example regions started to register rare diseases data in different periods: therefore, registrations identify both “historical cases” (cases whose information exists from a long time) and “new cases” and this may influence the quality of the NRRD and the precision of the estimates. Different approaches can be adopted to provide reliable incidence estimates; for example we may derive information on the registration probability for units in the population of interest, by looking at similar studies developed in comparable settings. When several registries are available, we may apply capture-recapture methods (see [2,3]): given a set of registries, we observe how many lists have registered the same unit. In this case, it is possible to assume that this value is a realization of a counting random variable with a known distribution (e.g. Binomial, Poisson, homogeneous or overdispersed, etc.). This distribution can be used to derive an estimate for the probability of not registering a generic unit.

Here, we aim at estimating the incidence rate for amyotrophic lateral sclerosis (ALS) in Italy, based on data derived from the NRRD only; the reason to focus on ALS is that it represents the second most frequent single pathology notified by the NRRD, after “hereditary coagulation disorders”. Due to privacy reasons, we could not consider other sources than the NRRD. In the following, we will show how to use the distribution of delay to ALS diagnosis to provide an estimate of the number of unregistered incident cases for the onset cohort 2007-2009.

## Methods

Incidence and prevalence rate estimates are often the main goals in epidemiological studies on disease distribution and the most difficult to get as well. Incidence conveys information about the risk of contracting the disease while prevalence indicates how widespread the disease is. Our aim is to give an estimate of the number of ALS incident units for 2007-09 in Italy starting from the cases registered in the NRRD. Due to the limited knowledge on the distribution of “risk” of diagnosis, we provide a non parametric estimate for the probability of a missing unit. The delay is calculated from the suspected disease onset (first referred symptoms) to the diagnosis for the disease. Thus, caution is needed since the date of first reported symptoms can be only a rough estimate of the true onset date; in particular, this date is based on individuals’ memory at the time of diagnosis. Therefore, it could be biased and the bias may increase with the distance from the first symptoms to the diagnosis date. The idea behind the proposed approach is that incident cases are only partially observed due to underreporting; that is, lack of a timely ascertainment may lead to a delayed diagnosis.

Potential reasons for late diagnosis include medical practice contributes: to enable earlier diagnosis, general practitioners should be informed of the usefulness of early referral for multidisciplinary care of patients, see [4,5].

We only observe people who have already been diagnosed, and therefore crude incidence rates will be probably downward biased for the most recent years since the observational window is too narrow.

Registries can be seen as endogenous mechanisms which identify *n* units from a population of (unknown) size *N* (in this context the “true” ALS incident population). The population size is given by the sum of the number of registered units *n* and the number of units which have not been registered by the mechanism/s *n*_0_. That is, the following equation holds

$$N=n+n_{0}$$

If a single registry is available, (the NRRD in this case) this empirical setting is described by the *N*-tuple (*δ*_1_,…,*δ*_*N*_), where *δ*_*i*_=1 if the i−th unit has been identified by the registry and *δ*_*i*_=0 otherwise. If the identification occurs independently for each unit with probability 1−*p*_0_, where *p*_0_ is the probability of not registering the unit, the conditional ML estimator of *N* is known to be the integer part of the Horvitz-Thompson estimator

$$\hat{N}=\left\lfloor \frac{n}{\left(1-p_{0}\right)}\right\rfloor$$

To get an estimate for *N*, *p*_0_must be known; otherwise, it should be estimated from observed data. As mentioned before, we adopt a non parametric estimator based on the distribution of the delay to ALS diagnosis. To define the estimator, the following assumptions are made: 

1. Conditionally on observed risk factors, all the cohorts are homogeneous, i.e. the distribution of the delay to ALS diagnosis does not vary over the observed time period (described by the cohort of onset).This means that the probability of experiencing the event “diagnosis of ALS” after a given time *t* from the onset is the same for individuals in the onset cohorts 2001-03, 2004-06, 2007-09. This is the most important assumption: if it holds, onset cohorts are “homogeneous” and we can use the information from older cohorts to estimate ALS incidence for the youngest one.

2. The registration of a subject does not depend on unobserved individual characteristics.This means that all individuals have the same proneness to be registered; therefore we may account only for observed heterogeneity trough considering variables in the NRRD, should these be reliable (i.e., compulsory and not voluntary etc.).

3. A generic unit may not be registered due, only, to delay to diagnosis (we do not consider ALS related deaths or migrations, i.e. the population is closed).This hypothesis holds only if the observational period is not too long, making negligible the probability of deaths, migrations etc. The occurrence of early death and/or faster disease course could influence the observed distribution of delay to diagnosis; also underreporting due to the lack for a definitive testing or possible mimicing of more common diseases may influence the observed distribution. As discussed by [6], ALS shows almost invariably a subtle onset, and this may lead to difficulties in early diagnosis, despite the growing number of clinical trials which undoubtedly encourage researchers to put much effort on a timely identification of the disorder. Previous studies have shown, in fact, little change in the diagnostic delay in ALS, with a median delay from onset to diagnosis of about 9–13 months, see also [5], which is clearly consistent with our data. Further factors that may influence a delayed diagnosis include mismanagement by general practitioners as well as by neurologists, see [6]. They have performed a logistic regression analysis which shows that initial misdiagnosis in ALS is unrelated to variables as gender, age at onset, diagnostic delay, site of onset, and being first observed by a neurologist. They suggest that cognitive errors, with particular reference to framing effects and anchoring heuristics may play a relevant role in the diagnostic delay in ALS. In fact, symptoms and signs of ALS can easily be framed in and anchored to more common pathological conditions, see also [7].

The statistical problem is to make inference about right truncated data, given that we condition on a given calendar time which represents the date the archive has been closed and data have been made available.

Solutions to this problem have been discussed, for example, by [8] who adjusted for reporting delay to provide a timely estimate of suicide incidence in Hong Kong; [9] present a parametric model for analysing the reporting delay for multiple sclerosis (MS) based on the approach discussed in [10].

An extensive literature treats about incidence and/or prevalence estimates (see for example [11-14]) especially in the context of AIDS, where the delay to diagnosis is projected back to get incidence estimates. Some studies are based on truncated failure time models, in semiparametric and non-parametric settings. Just to give an example, [15,26] aim at estimating the total number of HIV infected individuals: in fact only those who develop AIDS by a certain date are identified. They focus on situations where the AIDS is diagnosed is known and, at that time, the date of the initiating event (infection) is ascertained. The statistical problem is to make inferences about a stochastic process of infection and disease where realizations are right truncated in chronologic time. They consider the process in reverse time, transforming observed (right-truncated) data to survival data that are left-truncated in internal time; following their approach we use the ML estimator of the cumulative distribution function (cdf) *F*(*t*∣*τ*) of the delay between onset and diagnosis (conditionally on the truncation point), for a theoretical discussion see [9,15-17]. Here, the hypotheses are a bit different: we are interested in estimating the number of ALS people missed at registration because of delay to diagnosis, while [15,16] are interested in predicting the number of people infected (onset) but not reported yet in AIDS registries. Individuals who experience the first event do not necessarily experience the second one; in our formulation people experiencing the first event are supposed to necessarily experience the second one but they might not be observed due to a long delay to diagnosis (at least from a purely formal point of view). Deaths and censored data can not be observed. In the present context all observed cases are diagnosed or lost to the study due to death, a long delay to diagnosis, or failed ascertainment. [16] suggest to consider a bivariate process for inference, where the first process models the onset time and the other one describes the lag between infection and diagnosis. A bivariate Poisson process could be used also in the present context to simultaneously model the onset process and the time to diagnosis. However if a non parametric distribution is used for the process describing the arrivals, i.e. the onset times, the estimates described by equations (11) and (12) of [16] are identical to those proposed by [15] by looking only at the distribution of the delay to diagnosis regardless of the distribution used for the delay to diagnosis. The estimation approach developed by [15] is based on a truncated likelihood approach in reverse time, thus a relation between non parametric estimation based on the full likelihood and parametric estimation via the truncated likelihood (with a bivariate Poisson process) can be established. In our case a non parametric approach is preferred given that, unlike [18], we do not consider the whole observational period (e.g. 2001-2009) but are interested in estimating the incidence rate for ALS for the cohort with onset in 2007-2009 by using information from the older onset cohorts (2001-2003 and 2004-2006). Since information on onset cohort 2001-2003 and 2004-2006 is quite small due to the reduced sample sizes, we decided not to use a parametric approach for the arrival (onset) times, which could be an efficient and probably preferable approach, should we be able to select the appropriate parametric form for the corresponding distribution.

Given modeling assumptions, let *T* denote the random variable (rv) “time to diagnosis” and *t*_*i*_be the corresponding observed value for the *i*-th individual; furthermore, let *x*_*i*_be the time of disease onset for that individual. As mentioned before, *t*_*end*_ denotes the end of the observation period (say the upper bound of the time period covered by the available data, in the present case, 21 December 2009).

In the following, we will assume that the *i*-th subject would have not been registered should the corresponding diagnosis date be greater than *t*_*end*_, i.e. if *t*_*i*_ + *x*_*i*_>*t*_*end*_, see [19]. Thus the probability of being registered is

$$1-p_{0i}=\mathrm{Pr}(t_{i}\leq t_{\text{end}}-x_{i})=F(t_{\text{end}}-x_{i})=F(\tau_{i})$$

Following the retro-hazard approach proposed by [9], we project this probability from older cohorts to the youngest one to account for potential underreporting of cases with a more recent onset. This choice is motivated by the fact that, when considered alone, the 2007-09 onset cohort accounts for about 50% of the total number of registered cases; therefore, considering the whole period to estimate the cdf would over represent cases with shorter delays.

Given the introduced notation, *t* will be referred to as the reporting delay; according to [9] we assume that *X* and *T* are independent discrete random variables; these are observed only if *X* + *T*≤*t*_*end*_. Since we are interested in the distribution of *T*, we condition on the events (*X* + *T*≤*t*_*end*_) and (*X*=*x*) and define *τ*=*t*_*end*_−*x*. The retro hazard function can be estimated by the ratio

$$\hat{\rho }(u)=\frac{D_{u}^{*}}{R_{u}^{*}}\;u=1,\ldots ,\tau^{*}$$

where *τ*^∗^=*max*(*τ*_1_,…,*τ*_*n*_), *D*_*u*_={*i*∣*t*_*i*_=*u*} is the set of people who experience the diagnosis at time *u*, $\left(D_{u}^{*}=♯D_{u}\right)$, *R*_*u*_={*i*∣*t*_*i*_≤*u*≤*τ*_*i*_} is the set of people at risk (in backwards) at time *u*,and $\left(R_{u}^{*}=♯R_{u}\right)$. The part of the distribution function we are able to identify can be estimated by

$${\hat{F}}_{T}(t|\tau^{*})=\overset{\tau^{*}}{\underset{u=t+1}{\prod }}(1-\hat{\rho }(u))$$

see [9].

With a sufficiently wide time window i.e. with a sufficiently high *τ*^∗^, *F*_*T*_(*t*) can be well approximated by ${\hat{F}}_{T}(t|\tau^{*})$. This implies that *F*(*τ*)≃1; should not this be the case, ${\hat{F}}_{T}(t|\tau^{*})=\frac{{\hat{F}}_{T}(t)}{1-\theta }$ and, therefore, $\left(1-\theta ){\hat{F}}_{T}(t|\tau^{*})={\hat{F}}_{T}(t\right)$.

The retro hazard approach could be applied to the whole sample after stratifying according to potential risk factors such as gender. While a known gender effect in incidence of ALS has been elsewhere reported, see among others [20], the available data do not support differences in the distribution of the delay to diagnosis between males and females. The samples are substantially homogeneous both in size and for the retro hazard estimates; therefore, gender does not seem to play a significant role. See the discussion on this point in the exploratory data analysis section below.

By associating to each individual the value of the corresponding cdf estimate, i.e. the value at *t*_*i*_, we may approximate the probability of being registered until *t*_*end*_as

$$1-{\hat{p}}_{0i}=\hat{F}(t_{i})=(1-\theta )\hat{F}(t_{i}|\tau^{*})$$

and provide the following Horvitz-Thompson (HT) estimator

$$N=\overset{n}{\underset{i=1}{\sum }}\frac{n_{i}}{1-{\hat{p}}_{0i}}=\overset{n}{\underset{i=1}{\sum }}\frac{n_{i}}{\left(1-\theta )\hat{F}(t_{i}|\tau^{*}\right)}$$

where *n*_*i*_ identifies all units with the same onset time (in general *n*_*i*_=1), see [19].

The method is designed to deal with homogeneous populations where individual variables have similar effects on the probability of the event at a given time. This hypothesis is unlikely when dealing with real data where individuals differ for observable (i.e. age, gender, socio-economical status) and/or unobservable (i.e. different proneness to be registered) characteristics. In particular, when handling diseases, socio-economic variables may play an important role since they could be related to the timeliness in access to health services and in the disease ascertainment.

The presence of heterogeneity could be further investigated through a discrete time regression model which would allow to estimate the cdf conditionally on some covariates, see [9]; in this context, the current unreliability of socio economic variables in the NRRD (education and professional status) does not suggest to pursue this approach further, but we hope this could be done in a next future.

Another important issue in this context concerns mortality. The role of mortality is not negligible and related information would be useful to provide more precise incidence estimates; features specific to ALS disease led many researchers to use mortality to indirectly estimate incidence (see e.g., [21]: death percentages due to ALS as first death cause vary from about 34% to 92%. The relation between incidence and mortality is obviously bidirectional; a strong correlation between these two measures is present for the elderly. Given that the ALS diagnosis is difficult to be recognized in the elderly (due to the potential presence of comorbidities), this selection mechanism could lead to underestimate mortality; in this sense, survival analysis stratified by age or age groups would be important. The NRRD contains information about the date of death but this variable is not reliable since it is not compulsory and the corresponding information is only rarely recorded. Should the quality of mortality information be improved through permanent link with census archives, it could be possible to consider the observed (registered) removals to provide more precise incidence and prevalence estimates. In particular, it could be possible to register the number of people affected by ALS having a certain onset date but lost due to death/ migration and remove them to get more reliable prevalence measures. Should a link with Death causes survey (DCS) be possible, we could also try to understand how many incident units have not been registered by the NRRD but died of ALS, or with ALS registered as a comorbidity. This could help get a more reliable incidence measure as well. A probabilistic record linkage could be carried out between NRRD and survey of causes of death (DCS) by using appropriate matching variables; however this has not been possible yet due to privacy reasons (individual data can not be joined). Mortality can affect incidence and be affected by changes in health services which may lead to overestimation of death cases; improvements over recent years in ALS diagnosis may, at least hypothetically, explain the rise in death rates (see [21]) with ageing of the population.

As mentioned above, an important variable is the age at onset and the issue in this case is to verify whether it affects the delay to diagnosis and indirectly the incidence estimates. From an exploratory analysis, the percentage distribution of ALS cases for the following age groups “0-17”; “18-48”, “49-64”, “65-74”, “75+” has been carried out for each onset cohort (2001-03, 2004-06, 2007-09). The greatest percentage characterizes the class 49-64 in all the onset cohorts; in particular, this class weights about 44% for the onset 2001-03, about 36% for the cohorts 2004-06 and 2007-09. The age group 65-74 represents the second for size in all the onset classes (about 27%, 39% and 35% respectively). The percentages in the other age groups (0-17, 18-48 and 75+) are much less relevant, in particular the first group varies from 0% to 0.27% when 2001-03 and 2007-09 onset are considered.

**Figure 1:**
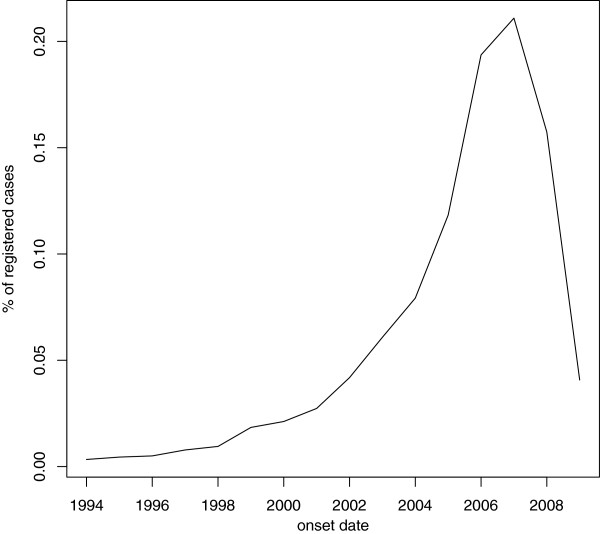
**Cdf estimates for delay to ALS diagnosis; cohort 2001-03 stratified by age at onset.** Time measured in days since first reported symptoms (onset). Legend: red line: Age [18,49) green line: Age [49,65) blu line: Age [65-75) black line: Age [75+).

**Figure 2:**
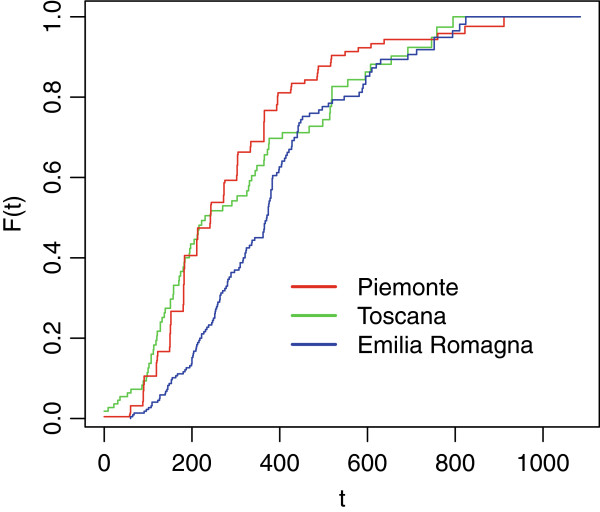
**Cdf estimates for delay to ALS diagnosis; cohort 2004-06 stratified by age at onset.** Time measured in days since first reported symptoms (onset). Legend: Red line: Age [18,49) Green line: Age [49,65) Blu line: Age [65-75) Black line: Age [75+).

**Figure 3:**
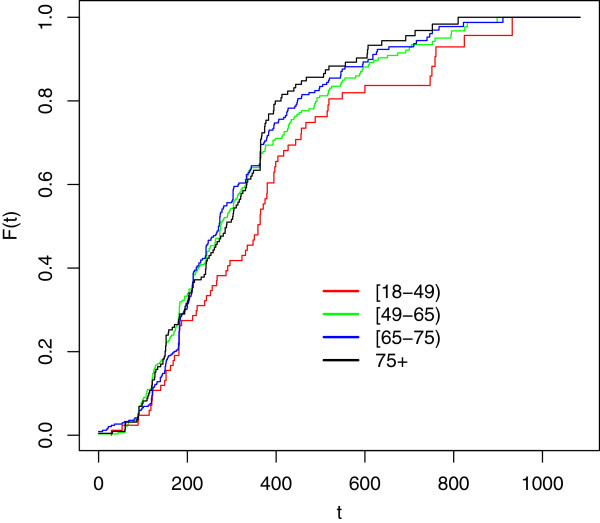
**Cdf estimates for delay to ALS diagnosis; cohort 2007-09 stratified by age at onset.** Time measured in days since first reported symptoms (onset). Legend: Red line: Age [18,49) Green line: Age [49,65) Blu line: Age [65-75) Black line: Age [75+).

Cdf estimates stratified by age at onset for each cohort are reported below (see Figure 1, 2 and 3).

As it can be noticed, as long as we consider the youngest onset cohort, characterized by greater sizes, the differences between cdf estimates by age at onset get smaller and smaller. This means that the age-specific estimates for each onset period are similar; this (approximate) homogeneity implies that age at onset does not seem to influence the delay to diagnosis for the considered data.

## Exploratory data analysis

Due to the registry quality, the following analysis are conducted considering only diagnoses occurred after 1993. The number of units who have experienced an ALS diagnosis between 1993 and 2009 is equal to 1799; if a unit has received the diagnosis twice, e.g. in two different presidiums or in two different Regions, only the first (less recent) diagnosis date has been retained. Only 26 units have been registered twice by the NRRD.

An higher portion of the 1799 registered cases are resident in Northern regions; 22*.*5*%* in Toscana, 21*.*68*%* in Piemonte and 13*.*67*%*in Emilia Romagna (see Table 1); southern regions are characterized by lower percentages, Sicilia 0*.*28*%* and Basilicata 0*.*89*%*(see Table 1).

**Table 1:** Number of registered ALS cases and population by Region of residence: details on Region sending ALS data to the NRRD (∗= Regions has sent data, −= Region has not sent data)

**Region of**	**Number**	**%**	**Population**	**Regions**
**residence**	**of cases**	**of cases**	**(%)**	**sending ALS**
				**data**
Abruzzo	9	0*.*50*%*	2,22*%*	-
Basilicata	16	0*.*89*%*	0,98*%*	*
Calabria	24	1*.*33*%*	3,33*%*	*
Campania	22	1*.*22*%*	9,65*%*	-
Estera	2	0*.*11*%*	-	-
Emilia Romagna	246	13*.*67*%*	7,28*%*	*
FVG	5	0*.*28*%*	2,05*%*	*
Lazio	195	10*.*84*%*	9,42*%*	*
Liguria	15	0*.*83*%*	2,68*%*	-
Lombardia	95	5*.*28*%*	16,28*%*	*
Marche	33	1*.*83*%*	2,58*%*	*
Molise	3	0*.*17*%*	0,53*%*	-
Piemonte	390	21*.*68*%*	7,37*%*	*
Puglia	148	8*.*23*%*	6,77*%*	*
Sardegna	3	0*.*17*%*	2,77*%*	*
Sicilia	5	0*.*28*%*	8,36*%*	-
Toscana	405	22*.*51*%*	6,18*%*	*
TAA	23	1*.*28*%*	1,70*%*	*
Umbria	18	1*.*00*%*	1,49*%*	-
ValledAosta	3	0*.*17*%*	0,21*%*	-
Veneto	139	7*.*73*%*	8,14*%*	*
Italy	-	100%	100%	

**Figure 4:**
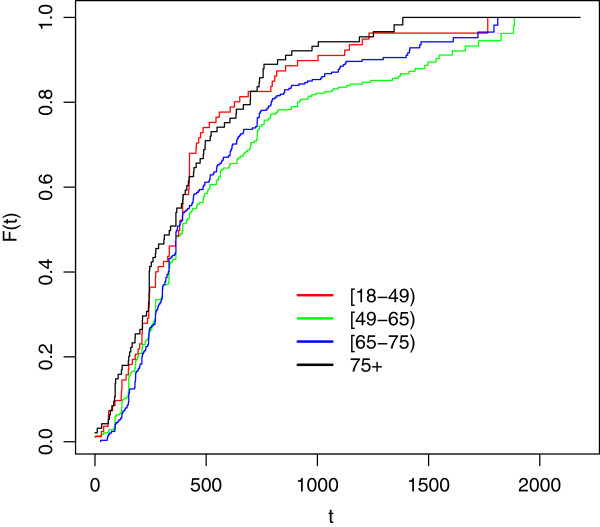
Number of observed cases by year of diagnosis, period 1993-2009.

According to the improvements in the ascertainment and in the registration procedures which have been observed in the last few years, the number of registered cases increases over time (see Table 2, Figure 4). The percentage of cases by year of diagnosis is 0*.*22*%*for 1993, 21*.*85*%*for 2007, 23*.*79*%*for 2008 and 18*.*29*%*for 2009. The number of cases registered in 2009 is lower than those in 2007 and 2008, highlighting the effect of underreporting due to delays in delivering data to the registry. Just to give an example, cases registered in the last semester of 2009 could not be uploaded yet at 21 December.

**Table 2:** Distribution of registered ALS cases by year of diagnosis

**Year of diagnosis**	**Number of cases**	**Percentage**
1997	12	0*.*67*%*
1998	11	0*.*61*%*
1999	12	0*.*67*%*
2000	34	1*.*89*%*
2001	31	1*.*72*%*
2002	37	2*.*06*%*
2003	71	3*.*95*%*
2004	85	4*.*72*%*
2005	122	6*.*78*%*
2006	223	12*.*40*%*
2007	393	21*.*85*%*
2008	428	23*.*79*%*
2009	329	18*.*29*%*

Considering the last years, the greatest percentages of ALS cases have been registered in Emilia Romagna (16*.*54*%*, 21*.*26*%* and 27*.*05*%* in 2007, 2008 and 2009 respectively) and Piemonte (26*.*97*%*, 16*.*12*%*, 23*.*40*%*respectively).

**Figure 5:**
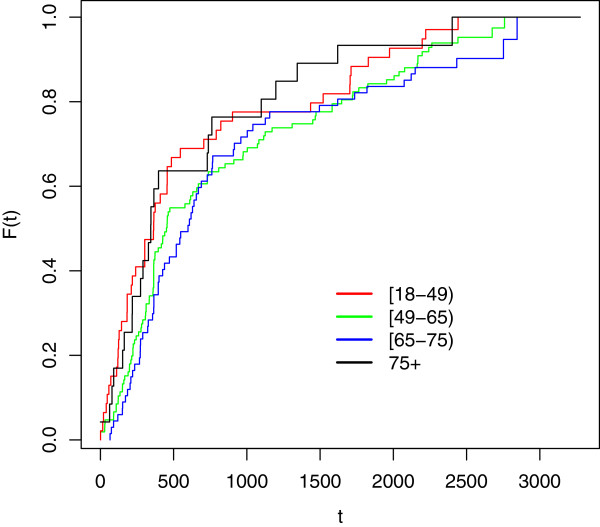
Number of observed cases by year of suspected onset, period 1993-2009.

As far as the onset date is concerned, 1*.*83*%* of total cases have reported a suspected onset in 1999, 21*.*01*%*in 2007 and 4*.*06*%*in 2009 (see Table 3, Figure 5).

**Table 3:** Distribution of registered ALS cases by year of onset

**Onset year**	**Number of cases**	**Percentage**
1994	6	0*.*33*%*
1995	8	0*.*44*%*
1996	9	0*.*50*%*
1997	14	0*.*78*%*
1998	17	0*.*94*%*
1999	33	1*.*83*%*
2000	38	2*.*11*%*
2001	49	2*.*72*%*
2002	75	4*.*17*%*
2003	109	6*.*06*%*
2004	142	7*.*89*%*
2005	212	11*.*78*%*
2006	347	19*.*29*%*
2007	378	21*.*01*%*
2008	282	15*.*68*%*
2009	73	4*.*06*%*

The decrease in the last years is more evident; registrations by year of onset decrease in 2008 and 2009 when compared to 2007. This evidence could be due to the effect of delay to diagnosis: incident cases for years 2008 and 2009 have not yet been registered. This could be better explained by looking at the distribution of observed delay to diagnosis (i.e. corresponding to already registered units). The whole sample is almost homogeneous: the size of the male sub sample is 980, while the female sub sample consists of 819 units. The mean delay to diagnosis is equal to 458 (se=11.53) days and agrees with data from international literature; other descriptive statistics are shown in Table 4. The median delay to diagnosis is equal to 316 days which corresponds to less than 1 year (around 10 months); the 25*th* percentile is 183 days (6 months) while the 75*th* percentile is 519 days (17 months). Tables 5–6 present summary statistics by gender; the median delay to diagnosis for females is equal to 334 days with 95% CI=(304;345), while the median delay to ALS diagnosis for males is equal to 306 days, 95% CI=(292;333). As it can be noticed by looking at the tables, the median delay for males is included in the 95% confidence interval of the median delay for females.

**Table 4:** Distribution of the delay to ALS diagnosis: percentiles, point estimates and relative CI (95%)

**Quantiles**	**Point estimate**	**95% CI (LB)**	**95% CI (UB)**
75	519	490	556
50	316	304	334
25	183	181	193

**Table 5:** Distribution of the delay to ALS diagnosis: percentiles, point estimates and relative CI (95%)

**Quantiles**	**Point estimate**	**95% CI (LB)**	**95% CI (UB)**
75	520	489	565
50	334	304	345
25	194	182	212

Female sample.

**Table 6:** Distribution of the delay to ALS diagnosis: percentiles, point estimates and relative CI (95%)

**Quantiles**	**Point estimate**	**95% CI (LB)**	**95% CI (UB)**
75	517	458	580
50	306	292	333
25	181	164	184

Male sample.

From now on, cases with unknown onset or diagnosis date will not be considered. The NRRD has been established in 2001 but contains data corresponding to previous time periods; for example,for ALS, the first known (registered) suspected onset dates back to 1987. However, only few cases (7% of the total number of registered cases) with onset before 2000 have been registered. The remaining 93% has been registered with the following time detail: 13% with onset in 2001-03, 39% in 2004-06 and 41% in 2007-09. We may argue that, before 2001, times to diagnosis were probably longer due to a less developed framework, to a reduced ability in recognizing the disease, to a lower quality of the health organization and to a lack of a unified framework for registering cases.

**Figure 6:**
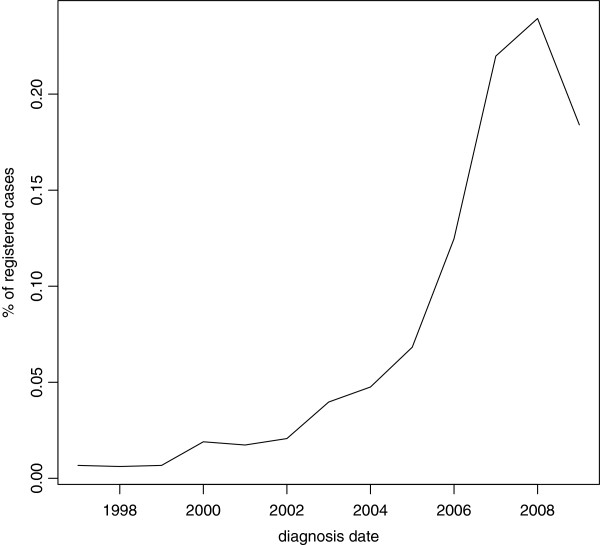
**Cdf estimates for delay to ALS diagnosis (2001-2009).** First 1000 days from the onset (time measured in days). LEGEND: Red line: Onset cohort 2001-03 Green line: Onset cohort 2004-06 Blu line: Onset cohort 2007-09.

The analysis has been conducted by stratifying the sample according to the time of suspected onset, defining three cohorts of onset: 2001-03, 2004-06, 2007-09. To assess whether a significant difference exists between the cohort-specific distribution of delay to diagnosis, we estimated the three cdf (one for each cohort) adopting the same length for the observation period; given that the cohort of patients with onset after 01/01/2007 has been observed until 21/12/2009, we estimated for all the cohorts the cdf of delay times corresponding to the first 1000 days from the beginning of the corresponding onset period. Figure 6 shows the estimates.

As it can be noticed by looking at the figure, the trends of the three curves are quite similar, they overlap in most of the points; however, the corresponding sample sizes are quite different and several ties can be observed at the end of the period.

A Kolmogorov-Smirnov (KS) test has been used to test for homogeneity of the cdf estimates. In particular, the cdf estimate for the most recent onset (2007-09) has been compared with the estimate for 2001-03 and 2004-06. While the first two functions (2007-09 vs 2001-03) do not seem to be significantly different (p-value=0.10), the cdf estimates for 2007-09 and 2004-06 seem to show substantial differences (p-value=0.0078). For the KS test comparing the cdf estimates corresponding to 2001-03 and 2004-06, the p-value is 0.17. The p-value for the comparison between the curve 2007-09 and the curve 2001-03 may be influenced by the differences in sample sizes (733 observation in 2007-2009 and 107 in 2001-2003).

The similarity between the cdf estimates stratified by onset cohort for the diagnoses within the first 1000 days from the beginning of the onset period, is the key result to produce a reliable ALS incidence rate estimate for the period 2007-09. In fact, conditional homogeneity of the different cohorts with respect to delay to diagnosis is the basic assumption beneath the proposed estimation approach. In this respect, our approach differs from that of [9]: while they used the whole time period (in the present case it would be 2001-09) to provide an estimate for the cdf, we feel this could lead to overestimate the weights associated to shorter delays since the size of the cohort 2007-09 is much greater than the previous ones. On the other hand, we build the weight on a few reported cases (for example those with onset in 2001-2003) and this could produce a higher variability in the resulting estimates.

**Figure 7:**
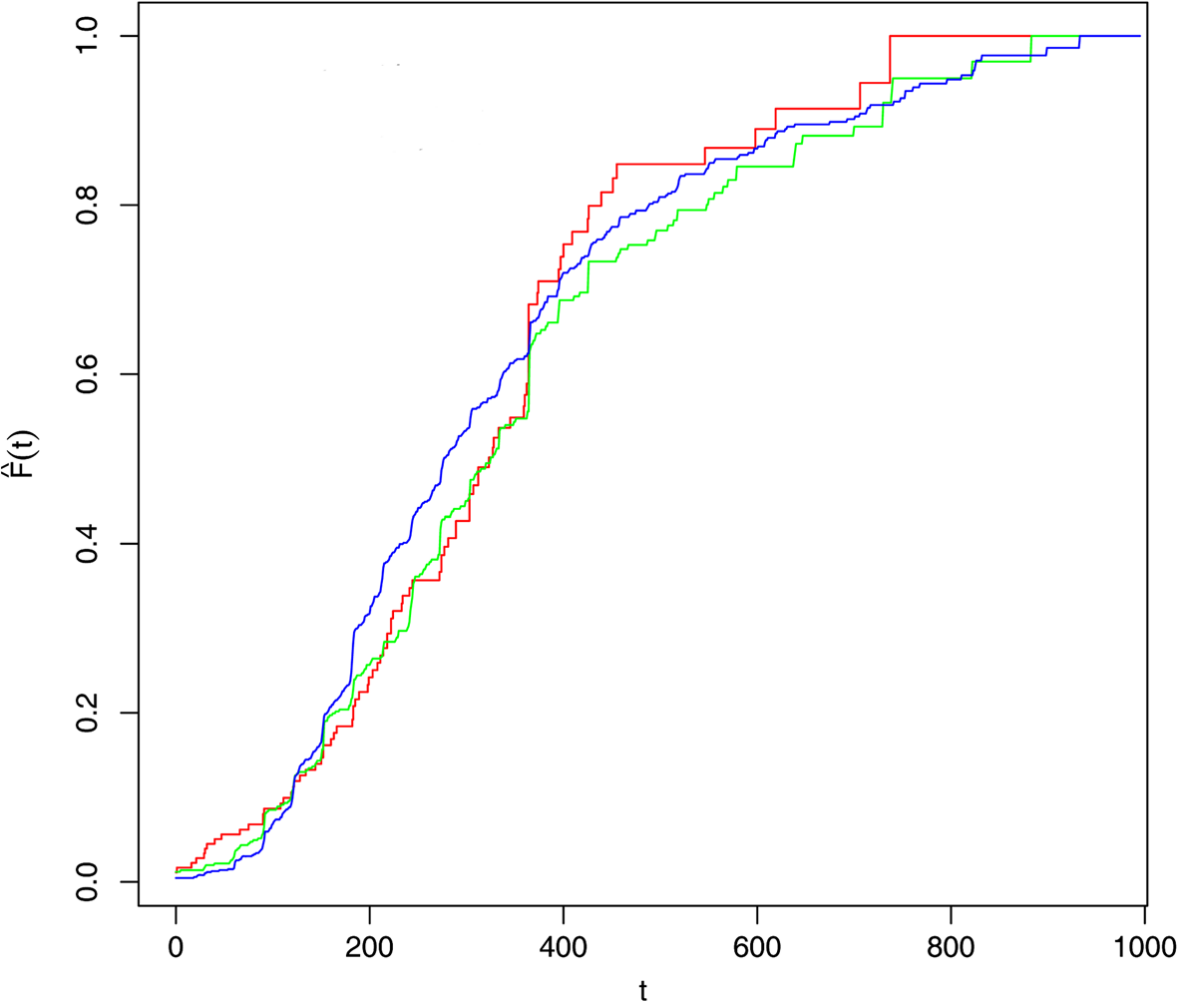
**Cdf estimates for delay to ALS diagnosis (2001-2009).** First 1000 days from the onset (time measured in days). LEGEND: Red line: Piemonte Green line: Toscana Blu line: Emilia Romagna.

Just to give additional insight on the geographical variability in the incidence rate estimates, we report in Figure 7 the cdf estimates for Piemonte, Emilia-Romagna and Toscana; these reagions account for around 58% of the total number of registered cases in 2007-09. Some differences do exist; however, they do not seem to be substantial even if the median delay to diagnosis for Emilia-Romagna is quite lower than the other two. This obviously points out the need to record richer information on newly registered cases since the proposed approach only accounts for a national behaviour in the delay to diagnosis distribution. As far as we know, the geographical variability in access to health services, in the health resources available to citizens as well as in the presence of highly specialized centers where more appropriate care pathways are established, could be a substantial factor influencing individual probability of being registered by the NRRD.

## Results

In this section, we show how current incidence rate crude estimates can be corrected for underreporting by using an Horvitz-Thompson (HT) approach. From the exploratory analysis, we saw that the cdf estimates referring to onset cohort 2001-03 is very similar to the one observed for 2007-09 when only patients with diagnosis within the first 1000 days from the beginning of the onset period are considered. This finding justifies the use of the HT approach detailed in section “Exploratory data analysis” to estimate the number of incident ALS units with onset in 2007-09; the individual probabilities of not being observed in the analyzed period have been estimated using the cdf estimate for the 2001-03 cohort. This onset cohort has been observed for a longer time (at least 6 years); for this reason we may assume that ${\hat{F}}_{2001-03}(t|\tau )≃{\hat{F}}_{2001-03}(t)$, i.e. *θ*≃0. We also give results based on the cohort 2004-06 estimate.

By using the cdf estimate from the 2001-03 cohort to weight the cases with onset in 2007-09, the HT estimator provides $\hat{N}=1344$ incident units, while the estimate obtained by considering the cdf estimate for the 2004-06 cohort is $\hat{N}=1206$ (obviously lower given that in the last case *θ*>0).

Since it is more likely that a higher part of incident cases with onset in 2001-03 have experienced the diagnosis before the end of 2009 as compared with 2004-06, and given the similarity between the cdf estimates for the cohorts 2001-03 and 2007-09, the HT estimate based on the cdf estimate for cohort 2001-03 may be considered as more reliable.

To assess the robustness of the proposed estimator, we applied this procedure to units with onset in 2001-2003 by weighting the cases registered before 21/12/2003, according to the approach described above, and compared this quantity with the number of units with onset in 2001-2003 and diagnosis date up to the 21 december 2009. In this way, we can see whether the underreporting observed in the first 1000 days from the beginning of the onset period can be due to delay to diagnosis. We used the same procedure for the onset cohort 2004-06 and found the following results. The estimated number of incident units with onset in 2001-03 is equal to 255, vs the observed 233; the estimated number of incident units with onset in 2004-2006 is equal to 744, vs 701 observed cases from the same onset cohort. The last comparison shows a greater difference which is probably due to the fact that the cohort with onset in 2004-06 has been observed for a shorter time period (when compared to the 2001-03 cohort). The resulting estimates are very similar to the observed counts, suggesting that the proposed procedure is adequate in estimating the number of units lost to the registration due to long delay to diagnosis; nevertheless this does not mean we are able to estimate the “total” number of units missed at registration due to other reasons, eg death, migration, uncorrect diagnosis, co-morbidities, etc.

To provide incidence estimates, we need to divide $\hat{N}$ by the corresponding population at risk; as previously mentioned, the NRRD is incomplete from a geographical point of view; in fact not all regions have sent rare diseases data to the national archive. In particular, for ALS, only information from 13 regions is available: Basilicata, Calabria, Emilia Romagna, Friuli Venezia Giulia, Lazio, Lombardia, Marche, Piemonte, Puglia, Sardegna, Toscana, Trentino Alto Adige and Veneto. Only 11 regions sent data where both onset and diagnosis dates are present (all but FVG and Sardegna). The corresponding number of person-year at risk amounts to 124,842,678 considering years 2007, 2008 and 2009 [22]; the observed crude ALS incidence rate calculated only through observed cases amounts to 0.59 per 100,000 people.

The annual ALS incidence rate estimated through the proposed approach is equal to 1.08 and 0.97 per 100,000 inhabitants considering the onset cohorts 2001-03 and 2004-06 as input for cdf estimates, respectively. The estimated 95% CI’s are (0.99;1.16) and (0.89;1.04) respectively. These results are not completely in line with those provided by the international literature, where annual incidence rates vary between 1.5 and 2.5 per 100,000 people.

## Discussion

The estimated incidence rates are however affected by a high regional variability in registration, as shown by the exploratory analysis. This means that underreporting might be due to delay to diagnosis and to geographical variability in reporting accuracy. If we do not consider Lombardia, given that the corresponding data come from local health unit registries and information related to onset and diagnosis dates are often not recorded, the estimated incidence rates for ALS are higher and closer to the international range: 1.32 and 1.19 with 95% CI’s equal to (1.21;1.43) and (1.09;1.29) as far as estimates from cohorts 2001-03 and 2004-06 are considered.

To verify the influence of geographical variability on incidence rate estimates, we calculated regional incidence rates through the same HT estimator, considering cdf estimates for cohorts 2001-03 and 2004-06. Results show very different values: in particular, incidence rates are higher in the northern regions and significantly lower in the southern regions of Italy.

Piemonte shows the highest incidence rate estimate: 2.43 per 100,000 people considering the 2001-2003 onset cohort and 2.25 per 100,000 as far as the cdf estimate from the 2004-2006 onset cohort is used. Emilia Romagna presents an estimated rate equal to 2.13 and 1.86 per 100,000 people for the two onset cohorts respectively. Southern regions show rates lower than 1 per 100,000: the rate estimates for Abruzzo are equal to 0.15 and 0.13 per 100,000; the estimates for Basilicata are 0.55 and 0.47, for Calabria 0.32 and 0.28 per 100,000 inhabitants.

These results confirm the existence of substantial differences in reporting accuracy, and point out where the system of data collection must be improved. In particular, by looking only at nothern Italy (Piemonte, Lombardia, Trentino Alto Adige, Veneto, Emilia Romagna) we get incidence rate estimates that are very close and even greater than the national ones; in particular they are equal to 1.24 and 1.11 per 100,000 inhabitants considering the 2001-03 and 2004-06 onset cohorts respectively. If we do not consider Lombardia, the incidence rate estimates in nothern Italy are higher: 1.89 and 1.70 per 100,000 respectively.

To give a comparison, a study performed in Piemonte and Valle d’Aosta during 1995-1996 by means of a prospective design estimated a mean annual ALS crude incidence rate of 2.5/100,000 population with a 95% CI=(2.2;2.9), see [23]. Few studies have comprehensively analyzed the time trend in ALS incidence; a prospective (population-based) study in Rochester, Minnesota, (USA) examined ALS incidence over time and showed that ALS incidence has been constant at 1.7 per 100,000 person year between 1925 and 1998. Increasing ALS incidence over time has been reported by other non-population based studies, but this could be due to improvements in case ascertainment and diagnostic methods rather than to a genuine increase in incidence, see [24]. A study conducted in Lombardia, see [25], where patients with newly diagnosed ALS were enrolled from 1998 to 2002 through a prospective regional register, revealed a standardized (with respect to age and gender) incidence rate of 2.09 per 100,000/year.

Recently, international and national studies focused on estimation of incidence rates stratified by gender and age etc.; for example, ALS incidence is known to be higher for males and at older ages. [24] reports ALS incidence rates in years 1988-1999 in selected studies with a prospective cohort design where age is adjusted to the 1990 US population and the age group 45-74 is considered. The rate estimates are equal to 5.2 in Scotland, 6 in Ireland, 5.5 in Washington, 5.4 in Piemonte and 4.2 in Puglia always per 100,000 person year.

## Conclusions

The aim of this study is to provide an estimate of the incidence rate for ALS (Amyotrophic Lateral Sclerosis) in Italy during the period 2007-09. ALS data are collected in the NRRD (National Registry of Rare Diseases) held by the ISS (National Institute of Public Health) with information on demographic, socio-economic and clinical characteristics. Due to low geographical and time coverage, registration quality, etc., the NRRD, like other pathology registries, is partially incomplete; a portion of incident ALS units has not been registered and needs to be estimated to achieve a reliable incidence rate estimate. Estimating the number of units missed at registration means estimating the probability of not registering a unit through the NRRD. Following [15,16,26] and [9], this probability is estimated through a reverse hazard model for the delay to ALS diagnosis, where delay represents the time intercurring between the onset and the diagnosis of the disease. The novelty consists in using information from cdf estimates corresponding to older onset cohorts (2001-03 and 2004-06) to give estimates for the youngest one (2007-09), after testing for homogeneity between the different cohort cdf estimates.

This approach is based on the assumption that a long delay to diagnosis may cause underreporting in the registry and influence the quality of the registry as well. The delay to diagnosis can also be linked to socio/economic status, age at onset, regional organization, etc. These determinants might be investigated through a proportional hazard regression model, see [10]; however, information available in the NRRD on socio-economic characteristics is not compulsory yet, therefore cannot be considered reliable.

In this case, we considered only the information on clinical variables and corrected the observed counts by providing an incidence estimate based on a HT type estimator for the number of ALS incident units. Incidence estimates are about 1 case per 100000 inhabitants and despite they let recovering a good part of underreporting, they are still far from ALS incidence international ranges between 1.5 and 2.5. This gap could be due to substantial regional variability in the NRRD: not all regions have sent data to the NRRD and not all regional presidiums are active in sending data as well. For these reasons, the population at risk would be lower and the estimated incidence rate could be higher, getting closer to the international values, as shown if we look at estimates for Northern Italy alone.

## Appendix A: Some more technical details

The conditional probability of observing an event exactly at time *t* is

$$\begin{array}{ll} f_{\tau }(t|\tau ,x) & =\mathrm{Pr}(T=t|X=x,X+T\leq t_{\text{end}})=\frac{f(t)}{F(\tau )}\\ & =f_{\tau }(t|\tau )\;0\geq t\leq \tau \end{array}$$

which depends on *x* only through *τ*.

As discussed by [15], we are only able to identify part of the distribution function, namely

$$F_{T}(t|\tau )=\frac{F(t)}{F(\tau )}\;0\geq t\leq \tau$$

which can be written also as

$$F_{T}(t|\tau )=\frac{f_{T}(t|\tau )}{\rho (t)}$$

where $\rho (t)=\frac{f(t)}{F(t)}$ is the retro-hazard or the reverse time hazard; it can be shown to be the same in the marginal distribution of *T* and in the distribution right truncated by *τ*. It follows that

$$F_{T}(t|\tau )=\overset{\tau }{\underset{u=t+1}{\prod }}(1-\rho (u))$$

The set {*ρ*(*t*)∣0≥*t*≤*τ*} describes the part of *F* that we can identify, see [15]. The likelihood function over *n* independent observations *t*_1_*t*_2_,…,*t*_*n*_ is given by

$$\ell (t)=\overset{n}{\underset{i=1}{\prod }}f_{T}(t_{i}|\tau_{i})=\overset{\tau^{*}}{\underset{u=1}{\prod }}\rho {\left(u\right)}^{D_{u}^{*}}{\left(1-\rho (u)\right)}^{R_{u}^{*}-D_{u}^{*}}$$

## Appendix B: Variance estimate for corrected incidence rate

As already mentioned, the crude ALS incidence rate *I*_*c*_for a given year is calculated as the ratio of the number *D* of new ALS cases to the person-time at risk, *S*, in the same period, *I*_*c*_=*D*/*S*. The proposed approach to adjust for the delay to ALS diagnosis is based on weighting each individual for the inverse of the probability of being observed and thus for the inverse of the cdf; in this way, each individual represents not only himself but also other units not registered. The proposed adjusted estimated incidence rate is given by

$$Î=\frac{\overset{n}{\underset{i=1}{\sum }}1}{1-{\hat{p}}_{0i}S}=\frac{\overset{n}{\underset{i=1}{\sum }}1}{{\hat{F}}_{i}S}=\frac{\overset{n}{\underset{i=1}{\sum }}{Ŵ}_{i}}{S}$$

*i*=1,…,*n* where ${\hat{F}}_{i}$ is the estimated cdf for the *i*-th individual and ${Ŵ}_{i}=\frac{1}{{\hat{F}}_{i}}$. The standard variance estimate of the crude incidence rate is (see [27])

$$v\hat{a}r(I_{c})=\frac{\hat{D}}{S^{2}}$$

In the present context, to estimate the variance of *I* we use the marginal variance rule

(1) $$\text{var}(I)=E_{S}[\text{var}(I|D,S)]+\mathrm{va}r_{S}[E(I|D,S)]$$

Using the idea that an estimate of *E*(*X*) is *X*, *E*(*var*(*I*|*D*,*S*) may be estimated using

$$\begin{array}{ll} E_{S}[\text{var}(I|D,S)] & =\text{var}\;\left(\;\frac{\overset{n}{\underset{i=1}{\sum }}W_{i}}{S}|D,S\;\right)\;=\;\frac{1}{S^{2}}\;\left(\;\overset{n}{\underset{i=1}{\sum }}\text{var}(W_{i})\;\right)\\ & =\frac{1}{S^{2}}\left(\overset{T}{\underset{t=1}{\sum }}\text{var}(W_{t_{i}})\right) \end{array}$$

Denoting with *D*_*u*_and *R*_*u*_ the number of observed individuals with ALS and the population at risk in the sample at time *u*, respectively, and knowing that (see [27])

(2) $$\text{var}(F_{t})={\left({\hat{F}}_{t}\right)}^{2}\overset{T}{\underset{u=t+1}{\sum }}\frac{D_{u}}{R_{u}(R_{u}-D_{u})}$$

According to [28], the delta method procedure help us approximate the variance of the transform $\frac{1}{F_{t}}$ as follows

(3) $$\text{var}(W_{t})=\text{var}\left(\frac{1}{F_{t}}\right)=\hat{\text{var}(F_{t})}{\left(\frac{-1}{{\hat{F}}_{t}^{2}}\right)}^{2}$$

plugging equation (2) into equation (3) we obtain

(4) $$\begin{array}{ll} \text{var}\left(\frac{1}{F_{t}}\right) & ={\left({\hat{F}}_{t}\right)}^{2}\overset{T}{\underset{u=t+1}{\sum }}\frac{D_{u}}{R_{u}(R_{u}-D_{u})}\frac{1}{{\hat{F}}_{t}^{4}}\\ & =\frac{\overset{T}{\underset{u=t+1}{\sum }}\frac{D_{u}}{R_{u}(R_{u}-D_{u})}}{{\hat{F}}_{t}^{2}} \end{array}$$

Furthermore, following [27] we can estimate *var*_*S*_(*E*(*I*|*NS*)) as

$$\mathrm{va}r_{S}[E(I|N,S)]=\overset{Â¯}{W^{2}}\hat{\text{var}(I_{c})}=\overset{Â¯}{{\left(\frac{1}{\hat{F}}\right)}^{2}}\frac{D}{S^{2}}$$

where $\overset{Â¯}{W^{2}}={\left(\overset{n}{\underset{i=1}{\sum }}W_{i}/D\right)}^{2}$

Thus, we obtain

$$\text{var}(I)=\frac{1}{S^{2}}\overset{T}{\underset{t=1}{\sum }}\left[\frac{\overset{T}{\underset{u=t+1}{\sum }}\frac{D_{u}}{R_{u}(R_{u}-D_{u})}}{{\hat{F}}_{t}^{2}}\right]+\overset{Â¯}{{\left(\frac{1}{F}\right)}^{2}}\frac{D}{S^{2}}$$

## Competing interests

The authors declare that they have no competing interests.

## Authors’ contributions

The manuscript is the product of the joint work of all the authors. However, Irene Rocchetti mainly contributed to sections *Methods*, *Exploratory data analysis* and *Results*, Domenica Taruscio mainly contributed to the *Background* section, while Daniela Pierannunzio mainly contributed to the *Conclusions* section, and *Results* together with the first author. All authors read and approved the final manuscript.

Consent

Written informed consent was obtained from the patient for publication of this report and any accompanying images.

## Pre-publication history

The pre-publication history for this paper can be accessed here:

http://www.biomedcentral.com/1471-2377/12/160/prepub
